# Machine learning-based 3D modeling and volumetry of human posterior vitreous cavity of optical coherence tomographic images

**DOI:** 10.1038/s41598-022-17615-z

**Published:** 2022-08-16

**Authors:** Hiroyuki Takahashi, Zaixing Mao, Ran Du, Kyoko Ohno-Matsui

**Affiliations:** 1 Department of Ophthalmology and Visual Science, Graduate School of Medical and Dental Sciences, Tokyo Medical and Dental University (TMDU), 1-5-45 Yushima, Bunkyo-ku, Tokyo, 113-8519 Japan; 2 R&D Division, Topcon Corporation, Tokyo, Japan

**Keywords:** Vitreous detachment, Eye manifestations, Three-dimensional imaging

## Abstract

The structure of the human vitreous varies considerably because of age-related liquefactions of the vitreous gel. These changes are poorly studied in vivo mainly because their high transparency and mobility make it difficult to obtain reliable and repeatable images of the vitreous. Optical coherence tomography can detect the boundaries between the vitreous gel and vitreous fluid, but it is difficult to obtain high resolution images that can be used to convert the images to three-dimensional (3D) images. Thus, the purpose of this study was to determine the shape and characteristics of the vitreous fluid using machine learning-based 3D modeling in which manually labelled fluid areas were used to train deep convolutional neural network (DCNN). The trained DCNN labelled vitreous fluid automatically and allowed us to obtain 3D vitreous model and to quantify the vitreous fluidic cavities. The mean volume and surface area of posterior vitreous fluidic cavities are 19.6 ± 7.8 mm^3^ and 104.0 ± 18.9 mm^2^ in eyes of 17 school children. The results suggested that vitreous fluidic cavities expanded as the cavities connects with each other, and this modeling system provided novel imaging markers for aging and eye diseases.

## Introduction

Vitreous is a large transparent gelatinous tissue that occupies the most of the eye volume. Vitreous contains metabolically active cells such as the hyalocytes, and it plays an important role in maintaining the homeostasis of the surrounding tissues including the sensory retina, ciliary body, and lens^1–4^. A degradation of the vitreous gel results in an increase of vitreous fluid and is accelerated with increasing age, intraocular inflammation and trauma^5–7^.

The structural changes of the vitreous gel have been studied in autopsy eyes. In 1977, Worst injected Indian ink into the vitreous of enucleated human eyes and showed that there were numerous cisterns within the vitreous gel^8^. However, the microstructures of the fluidic cavities were difficult to examine because human vitreous begins to degrade immediately after death.

Real-time imaging of the vitreous has been done by ultrasonography and magnetic resonance imaging (MRI). These methods have the advantage of imaging the entire extent of the vitreous, but their resolution is too low to differentiate the vitreous gel from the vitreous fluid.

Optical coherence tomography (OCT) is a non-contact, non-invasive, micron-scale optical imaging technology, and it is used as a standard diagnostic instrument by ophthalmologists. Swept-source/Fourier domain OCT (SS-OCT) can detect the boundary between vitreous gel and vitreous fluid because it can record images at a speed of 100,000 axial scan per second with an axial resolutions of 5 to 7 µm^9–11^. Thus, high resolution cross-sectional images of the posterior vitreous can be obtained by averaging multiple scans, and these show the fluidic cavities in the vitreous gel and the connections between cavities that are compatible with Worst’s observations. In addition, previous studies reported that premacular vitreous pockets (PMPs) had a flattened bowl-like shape^12,13^ and there is the connection between PMP and Cloquet’s canal (CC)^14,15^. However, structure of posterior vitreous is difficult to be understood only with cross-sectional images and obtaining reliable three-dimensional (3D) images of the vitreous gel is still not possible because the vitreous gel is continuously moving along with ocular movements. Thus, a cross-sectional image of a specific location can differ at different times.

This difficulty has been addressed by implementing machine learning (ML) techniques which are able to perform complex tasks of analyzing medical images accurately by employing multilayered artificial neural networks trained on a large data set of labeled images^16,17^. ML combined with OCT has been used in ophthalmology in various ways such as noise reduction of the images and automatic diagnosis of eye diseases^18–23^.

**Figure 1:**
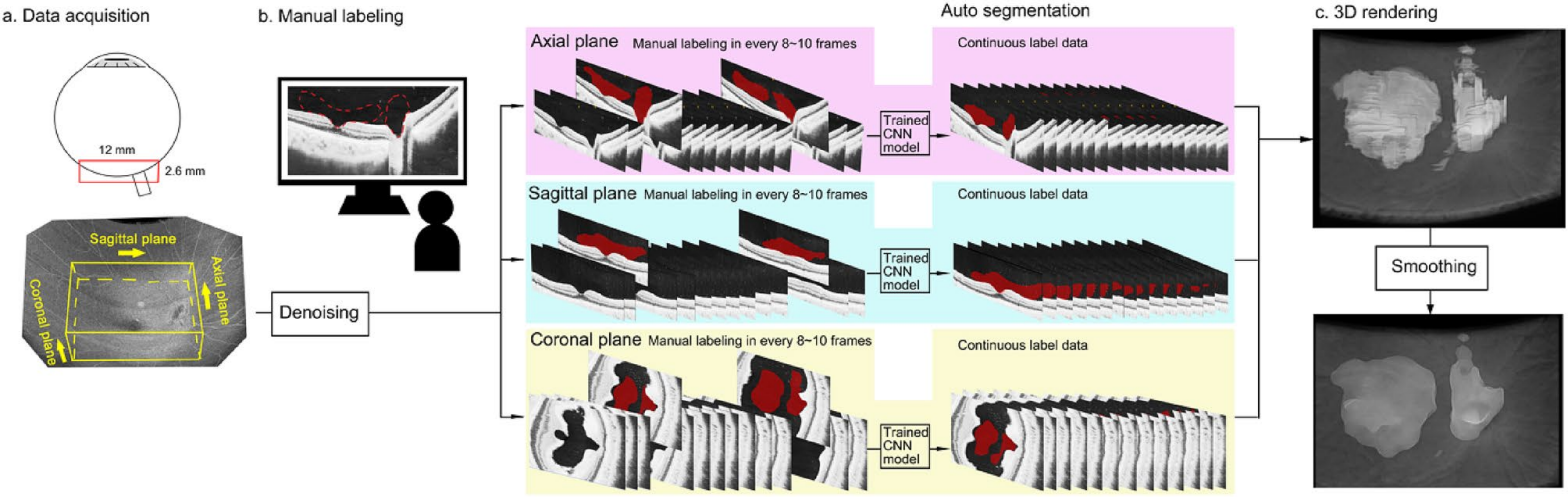
Machine learning-based detection and segmentation of vitreous fluidic cavities for 3D modeling of the posterior vitreous. (**a)** Optical coherence tomographic (OCT) volume scan data are acquired at the midpoint between the fovea and optic disc. The scanned area is set to 12 × 9 × 2.6 mm^3^ and orthogonal 3 planes of 2D OCT images are defined as axial, sagittal, and coronal plane. The OCT data are preprocessed with a deep learning (DL)-based noise reduction algorithm. (**b)** The vitreous fluid area was manually labelled in representative 2D OCT images for every 8–10 frames, along the axial, sagittal, and coronal planes. A deep convolutional neural network (DCNN) was trained to segment the vitreous fluid area based on the manual labels in each plane. Next, the trained DCNN was used to annotate the vitreous fluid into frames without manual labels. (**c)** A series of continuous labeled vitreous fluid is generated by combining all three planes. The 3D image was rendered and its brightness and contrast were adjusted with ImageJ. The 3D median filter with size of 9, 9, 9 was applied for the predicted 3D models to further reduce the noise and rough edges of the cavities.

We have recently developed a 3D modeling method using ML-based automatic labelling. With this method, we were able to determine the 3D structure of the posterior vitreous as static images^24^. An overview of the technique is presented in Fig. 1. These 3D models showed that the vitreous fluidic cavities were connected with each other complexly and were occasionally joined together to form a large fluidic cavity.

The consistency of this 3D model of fluidic cavities in the vitreous cavity was evaluated by calculating inter-rater reliability of 6 pairs of two 3D vitreous model for which two independent examiner made manual labels. In addition, we reviewed the data of 17 elementary school children with moderate myopia. The eyes of children were selected to measure the volume and surface area of each vitreous fluidic space such as the premacular vitreous pocket (PMP) and Cloquet’s canal (CC) in the vitreous mainly because these fluid spaces have no or little connection with each other and cisterns were not formed. Based on these measurements, we investigated the relationship of the shapes, volumes, and surface area in the 3D models.

## Methods

### Participants

All participants were recruited from the Tokyo Medical and Dental University Hospital. All procedures were performed according to the tenets of Declaration of Helsinki after approval of the protocols by the Institutional Review Board of Tokyo Medical and Dental University, Faculty of Medicine (Approval number, M2020-170). All participants received detailed explanation and were allowed to decline their participation any time. All participants provided written informed consent. For participants who are minors, informed consent was obtained from a parent. Detailed information is present in Japanese at the link of web site; https://www.tmd.ac.jp/bioethics/.

All participants underwent a comprehensive ophthalmic examination including an objective measurement of the refractive error using autorefractor (KR7100P, Topcon, Tokyo, Japan) without pupil dilation, and the axial length (IOLMaster, Carl Zeiss Meditec, Inc, Dublin, CA, USA). Twenty-four elementary school children who had no eye diseases except for moderate myopia were studied. The demographics of the children are shown in Supplemental Table 1. Patients with poor image quality due to unsteady fixation and indistinguishable boundary between vitreous gel and vitreous fluid on cross-sectional OCT images were excluded.

### OCT imaging acquisition and processing

Optical coherence tomographic data were recorded using a Topcon Triton SS-OCT (Topcon, Tokyo, Japan) with a 100,000 Hz optical beam repetition rate and with a laser light source with a central wavelength of 1050 nm. The extent of the scanned area was 12 mm × 9 mm and it was centered at the midpoint between the fovea and optic disc. The depth of the scanned area was 2.6 mm, and retina of patients was fixed at the center of scanned field with manual focus adjustment to maximize the quality of the posterior vitreous. Accordingly, the net depth of scanned posterior vitreous was about 1.2 mm.

### Denoising OCT images

All OCT data were preprocessed with UNet-based noise reduction algorithm as reported in previous study^25^. In this algorithm, a single cross-sectional OCT image was used for training instead of averaging multiple scans.

### Vitreous fluidic cavity segmentation and 3D modeling

Briefly, A segmentation of the vitreous fluid was performed using residual versions of the U-Net architecture in 3D. Each input data consisted of a 3D stack of the frame before the frame of interest, the frame of interest and the frame after the frame of interest. The additional information from the frame before the frame of interest and the frame after the frame of interest was used by the AI system to improve signal-to-noise ratio, and the methods were used as presented in detail^24^. All restoration experiments were performed in Python using TensorFlow. First, the vitreous fluidic area was initially manually labeled in representative 2D OCT images, e.g., every 8–10 frames, along the axial, coronal, and sagittal planes by retinal specialists (HT or KOM). Second, a deep convolutional neural network (DCNN) was trained to segment the vitreous fluid areas for each scan based on the said manual labels in each plane. Third, the DCNN was used to segment the vitreous cavity into frames with no manual labels. The complexity of the DCNN models was reduced by setting the depth of each convolutional filters to 32 and the learning rate was set to 0.0002 to achieve a slow and smooth convergence. Fourth, a series of continuous labels of vitreous fluid were generated by combining the three planes. For disagreements of the vitreous fluid among the planes, the final decisions were determined by the majority. Then, AI segmentation along the three planes (axial, coronal, and sagittal) produces three segmentation results for a voxel. In the end, a voxel is determined to be part of the vitreous pocket if at least two of the three segmentation results identify it as part of the vitreous pocket. Finally, we generated binary segmentation results of 3D vitreous cavity and the results are rendered by the ImageJ software (National Institute of Health, Bethesda, MD). The 3D median filter with size of 9, 9, 9 was applied for the predicted 3D models to further reduce the noise and to smooth the rough edges of the cavities. As the accuracy of AI segmentation and 3D modeling have been reported previously^24^, they were not reassessed in the present study.

### Biometry of volume and surface area in 3D vitreous cavity model

The volume and surface area of the 3D vitreous cavity were automatically calculated using the Python and the Scikit-Image library^26^. First, the predicted 3D vitreous cavities were scaled so that each voxel corresponded to a cube of 1.03 × 10^–4^ mm^3^. Then, the volume of the vitreous cavity was calculated by counting the number of voxels. Finally, the surface area of the vitreous cavity was calculated based on the voxels using the Marching Cube algorithm^27^.

### Statistical analyses

The statistical analyses were performed using SPSS version 24.0 software (SPSS, Chicago Illinois, USA). Data are presented as the means ± standard deviations (SDs). The significance of the difference between two groups was determined by Student’s *t* tests after their normal distributions were confirmed by Shapiro–Wilk tests. Cronbach’s alfa coefficients were used to evaluate the inter-examiner reliability for quantitative data of the 3D vitreous models. A probability (*P*) value of < 0.05 was considered significant.

## Results

**Figure 2:**
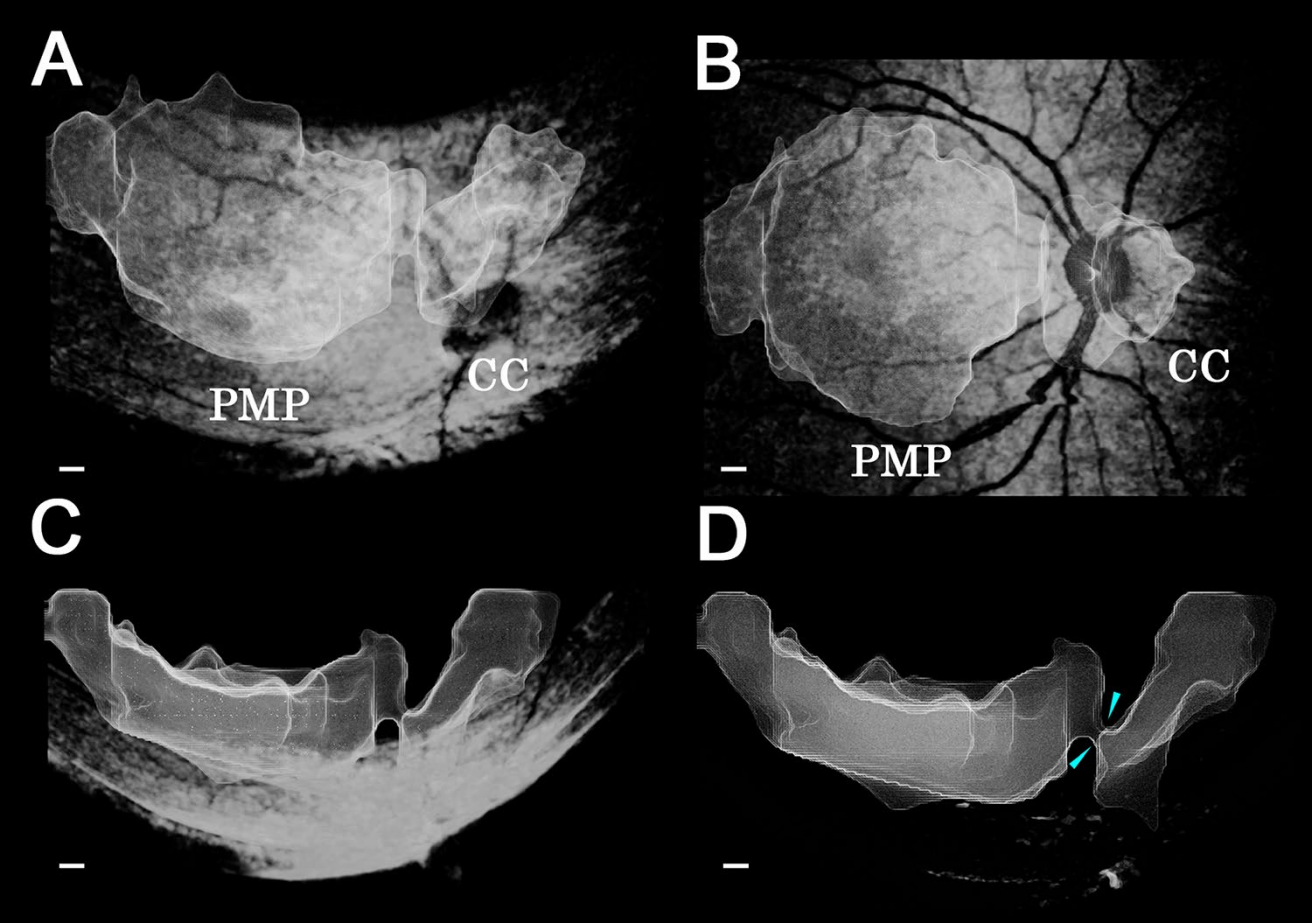
Three-dimensional model of the vitreous cavity of the right eye of a healthy 8-year-old girl. (**A)** Top view image from the inferior side of the eye shows that a premacular vitreous pocket (PMP) and Cloquet’s canal (CC) are present above the macular retina and optic disc. (**B)** Front view image of the 3D model shows that the PMP extends between the retinal vascular arcade. The volume and surface area of the PMP are 12 ± 1 mm^3^ and 75 ± 5 mm^2^ respectively. The CC is located anterior to the optic disc and the volume and surface area of the CC are 2.3 ± 0.1 mm^3^ and 19 ± 1 mm^2^ respectively. (**C**) Image viewed from the inferior side shows that the PMP and CC stand on the retinal surface. (**D)** Image viewed from the inferior side without the retinal image. Light blue arrowheads show the connection between the PMP and CC. The scale bar is 500 µm. Plus-minus values are the propagating error of the segmentation. *PMP* premacular vitreous pocket, *CC* Cloquet’s canal.

The 3D models of the posterior vitreous cavities were generated successfully from the SS-OCT 3D volume data with the aid of our labelling algorithm (Supplemental Movie S1, S2). The front view images of the 3D model showed that there were two distinct fluid cavities in the vitreous cavity located above the macular area and the optic disc. These two cavities corresponded to the PMP and CC (Fig. 2A,B). When the 3D model was viewed from a superior or inferior view of the macula area, the presence of a connection between the PMP and CC could be seen (Fig. 2C,D).

### Inter-examiner reliability

We manually labeled images to train the DCNN and hypothesized that trained DCNN depended on the examiners’ ability to annotate data. To evaluate their accuracy, we obtained 6 pairs of two 3D vitreous models for which two independent examiners (HT and KOM) performed manual labelling of each eye independently. The volume and surface area were measured for each model and inter-examiner reliability was analyzed.

The results showed that the Cronbach’s alfa coefficients were 0.92 and 0.82 for their volume and surface area respectively. The concordance between the 2 examiners was good for the measurements of both the volume and surface area of the vitreous cavities.

### Premacular vitreous pocket and Cloquet’s canal in eyes of elementary school children

**Figure 3:**
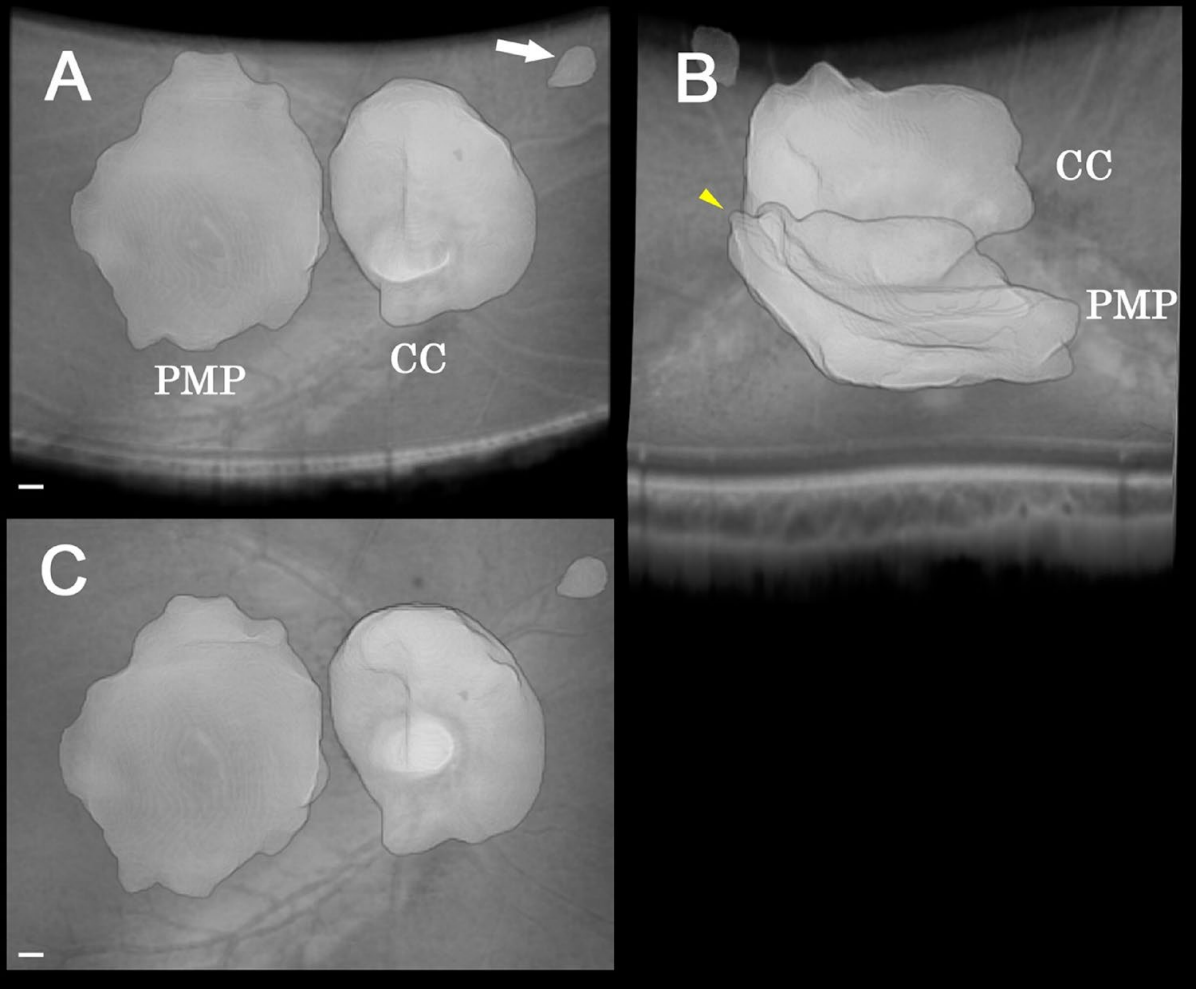
Three-dimensional vitreous cavity model of the right eye of a healthy 8 year-old boy. (**A)** Top view image from the inferior side of the eye. Fundus shows that the premacular pocket (PMP) and Cloquet’s canal (CC) are present above the macular area and the optic disc. Threre is another small cystic cavity at the area superior nasal to the macula (white arrow). (**B)** Image viewed from temporal side showing that the superior boundary of the PMP is lifted up anteriorly (yellow arrowhead). (**C)** Front view image of 3D model shows that the PMP extends between the retinal vascular arcade, and the superior boundary of the PMP lies over the superior retinal arcade. The volume of the PMP is 4.1 ± 0.2 mm^3^ and the surface area is 44 ± 3 mm^2^. CC is located in front of the optic disc. The volume of the CC is 5.2 ± 0.3 mm^3^ and the surface area is 33 ± 2 mm^2^. The scale bar is 500 µm. Plus-minus values are the propagating error of the segmentation. *PMP* premacular vitreous pocket, *CC* Cloquet’s canal.

Among the 48 eyes of 24 elementary school children with moderate myopia, 4 eyes were excluded because of poor fixation and 27 eyes because the boundary between the vitreous gel and vitreous fluid could not be detected. In the end, 3D vitreous models were generated for 17 eyes of these schoolchildren. The PMPs of the school children had a plate-like shape, and the superior boundary of the PMP was lifted anteriorly more than inferiorly in 11 of the 17 eyes (65%) viewed from the temporal side (Fig. 3B). In the other 6 eyes, the PMP stood symmetrically above the retina.

The volume and surface areas of the PMP, CC, and the entire cavities were measured for each 3D vitreous model. The mean volume of the PMP of 17 eyes was 16.08 ± 7.81 mm^3^, of the CC was 3.02 ± 1.28 mm^3^, and of the entire eye was 19.62 ± 7.80 mm^3^. The mean surface area was 82.03 ± 20.53 mm^2^ for PMP, 21.87 ± 6.76 mm^2^ for CC, and 104.04 ± 18.85 mm^2^ for the entire cavities (Table 1). There was a significant correlation between the volume and surface area of the PMP (R = 0.90; *P* < 0.001), CC (R = 0.91; *P* < 0.001), and the entire cavity (R = 0.82; *P* < 0.001).

**Table 1:** Clinical variables and biometric data of school children.

	Mean ± SD	Range
Age (years)	8.47 ± 1.14	7 to 10
Sex (male/female)	5/12	
Refractive error (diopters)	−2.25 ± 1.08	−1.00 to −4.38
Axial length (mm)	24.24 ± 0.88	22.40 to 25.96
**Volume (mm**^**3**^**)**		
Whole cavities	19.62 ± 7.80	6.91 to 33.95
Premacular vitreous pocket	16.08 ± 7.81	4.09 to 29.25
Cloquet’s canal	3.02 ± 1.28	1.35 to 5.60
**Surface area (mm**^**2**^**)**		
Whole cavities	104.04 ± 18.85	67.49 to 132.27
Premacular vitreous pocket	82.03 ± 20.53	43.74 to 113.26
Cloquet’s canal	21.87 ± 6.76	10.51 to 35.52

*SD* standard deviation.

Next, we investigated how each cavity correlated with the entire cavity for volume and surface area. The volume and surface area of the entire cavities were correlated with the volume and surface area of the PMP (R = 0.98, *P* < 0.001; and R = 0.90, *P* < 0.001), but there was no significant correlation of the volume and surface area between the entire cavities and CC (R = 0.30, *P* < 0.24; and R = 0.38, *P* = 0.14).

Among the 17 eyes of the school children, the age, refractive error, and axial length were not significantly correlated with the volume and surface area of the PMP, the CC, and the entire cavity. There were no significant differences in the volume and surface area of the PMP, CC, and the whole cavities between the eyes of boys and girls.

### Connections between premacular vitreous pocket and Cloquet’s canal

To determine whether there were connections between the PMP and CC, we examined 17 vitreous models of the school children. Connections were found in 14 eyes (82%). There were no significant differences in the age, sex distribution, refractive error, and axial length between eyes with and without connections. The mean surface area of the vitreous cavities was significantly larger in eyes with than without connections (108.64 ± 17.56 mm^2^ vs 82.56 ± 3.61 mm^2^, *P* = 0.03). On the other hand, the volume of the vitreous cavities was not significantly different between eyes with and without connections (Supplementary Table 1).

## Discussion

Recent developments in deep learning technology have enabled accurate automatic segmentation of retinal OCT images using different variations of the U-Net^28^. Dilated-Residual U-Net (DRUNET)^29^ was developed to segment various regions in OCT images including the retina, choroid and optic nerve head. ReLayNet^30^, a U-Net-based neural network developed for retinal layers and retinal fluid segmentation. Our U-Net-based 3D modeling showed that it is possible to obtain a single posterior vitreous model for each eye. To the best of our knowledge, this is the first time that ML-based 3D vitreous models was generated from 3D OCT data and quantified in a case–control way. The measured volumes and surface areas showed moderate inter-examiner reliability because each DCNN was trained with manually labelled segmentation data.

The 3D vitreous models allowed us to measure the volume of the vitreous fluidic cavities. Alterations of the posterior vitreous probably occurs during young adulthood when the retina and choroid show no evident age-related change. The posterior vitreous detaches from the inner retinal surface in the second decade of life when the retina and choroid do not show any signs of aging^31^. We have reported that the posterior vitreous thickened after a posterior vitreous detachment in a 27-year-old highly myopic woman^32^. Thus, quantification of the vitreous fluidic space might enable us to detect earlier manifestation of aging than that from other ocular tissues. In addition, structure of vitreous gel and fluid is reported to be altered in eyes with diabetic retinopathy, myopia, and intraocular inflammation before patients develops the symptoms^33,34^. Then, biometric data of vitreous is a possible indicator for subclinical changes of the diseases.

Our results showed a significant correlation between the volume and surface areas of the vitreous fluidic cavities. This suggested that the shape of the cavities might be similar among eyes even though the absolute size varies. In addition, the entire volume of the vitreous fluidic cavities was significantly correlated with the volume of the PMP but not with the volume of the CC in each eye. These findings suggested that the expansion of the fluidic spaces resulted mainly from an expansion of the PMP rather than the CC. Neither the volume nor the surface area of the vitreous was significantly correlated with the age, sex, and axial length in our cohort. Earlier, the weight and width of the PMP were measured in the cross-sectional OCT images, and they were reported to be correlated with the age and axial length^10^. Although the relationships among the height, width, volume, and surface area of the vitreous were not evaluated, the small number of subjects possibly affected our results.

On the 3D images, the superior edge of the PMP were closed and lifted up in 65% of the children (Fig. 3). There is difference of the shape of PMP between children and adults whose PMPs were not limited to a single isolated cavity, but they were connected with adjacent vitreous cisterns by trunk-like structures at the superior edge in our previous study^24^. These observations suggest that the vitreous cisterns and their connections are premature if found in childhood.

Our results showed that 14 of 17 (82%) eyes had a connection between the PMP and CC and there were no significant differences in the age, sex, axial length, and refractive error between eyes with and without connections. The prevalence of the connection has been reported to be 17 to 22% and be associated with age and axial length in earlier studies^14,15^. Although our 3D vitreous modeling might improve the sensitivity of the detection of the connections by viewing the images from multiple sides, the relationship between age, axial length and the presence of connection could not be determined because our cohort was smaller and older than previous studies.

The mechanism for the development of the connections between PMP and CC has not been definitively determined. In a previous study, cracks in the premacular vitreous gel were found in the cross-sectional OCT images of a 3-year-old girl but not in images of a 2-year-old boy^35^. Our results showed that the surface areas of vitreous fluidic cavities were larger in 14 eyes with a connection between PMP and CC than in the three eyes without a connection. These observations suggest that vitreous fluidic cavities expand as the connection between PMP and CC matures.

**Figure. 4:**
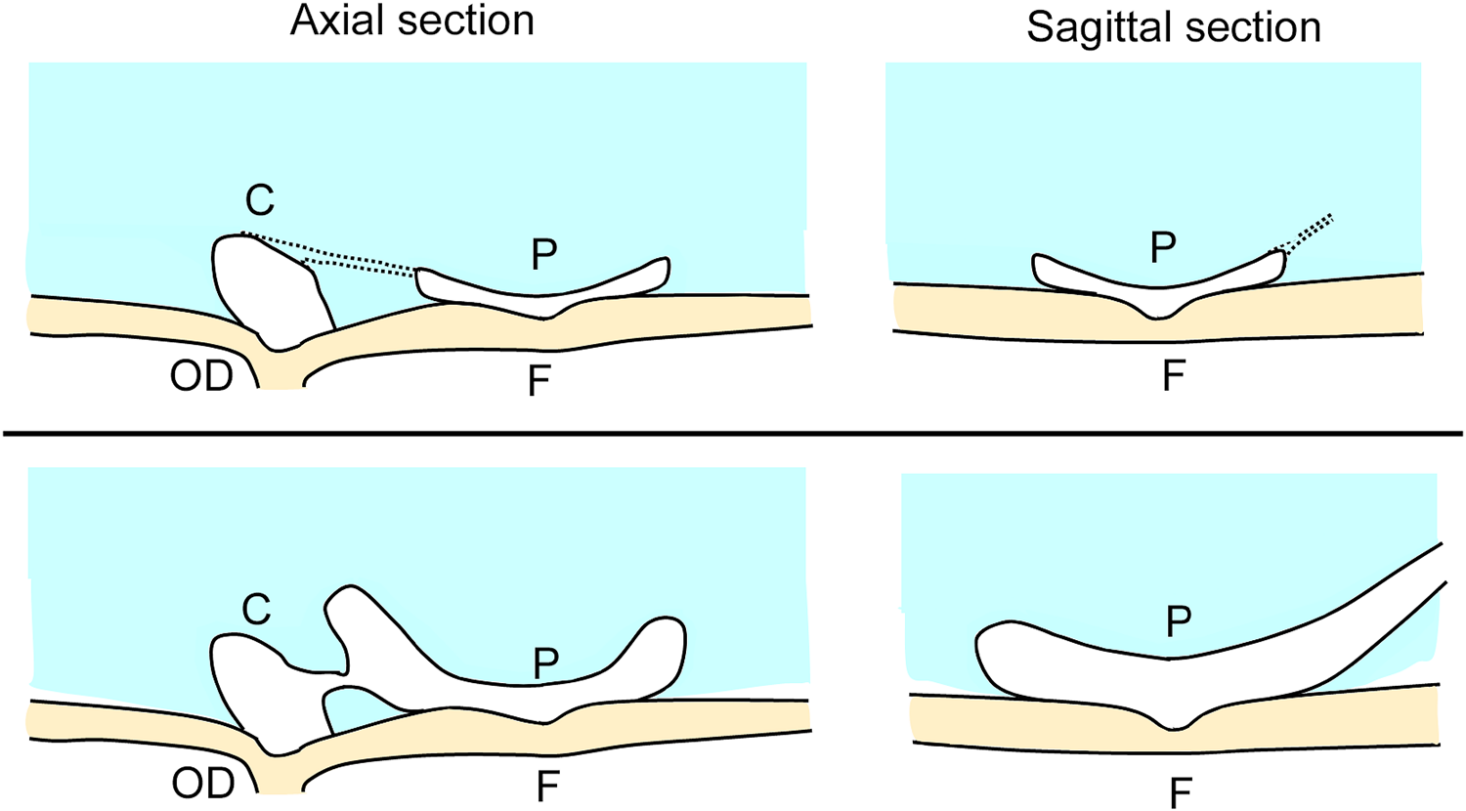
Schematic diagram showing the course of the expansion of the vitreous fluidic cavities and the development of connections between the cavities. Top row: at birth, Cloquet’s canal (CC) and a small premacular vitreous cavity are present on the retinal surface. These cavities are connected by narrow canals as seen in the axial sectional image. On the sagittal section image, the superior boundary of the premacular vitreous pocket (PMP) is lifted-up and the narrow canal extends anteriorly. Bottom row: with increasing age, the fluidic cavities expand and the PMP develop. The connection between PMP and CC is also seen in the axial sectional images. In the sagittal sectional image, trunk-like fluidic space can be seen to expand anteriorly from the superior edge of the PMP. *C* Cloquet’s canal, *P* premacular vitreous pocket, *F* Fovea, *OD* optic disc.

The process of the growth of vitreous fluid has not been investigated well in school children. Based on the findings in our 3D vitreous models, a schematic diagram shows how the vitreous fluidic cavities expand along with the development of the connections between the cavities. At birth, the CC and small premacular cavities are present on the retinal surface, and they are connected by a narrow canal, a remnant of the hyaloid vascular system. With increasing age, these canals enlarge and form the connection between cavities as the vitreous fluid cavities expand (Fig. 4).

The limitations of our study mainly lie in the relatively small number of 3D models. Thus, we do not have strong evidence that our results are also present in the general population. Second, our 3D vitreous model was not applied to adult eyes in which the vitreous is more liquefied and disorganized than those of children and young adults. Even in school-age children, manual labelling could not be applied in 31 eyes (65%) due to poor quality of the cross-sectional OCT images. Third, 3D vitreous model was generated only once for each eye, and follow-up observations were not performed. Thus, our suggestion on the expansion and development of vitreous fluidic cavities is based on a small quantity of data. Consequently, determining the full range of applicability of this technique requires longitudinal studies with a larger number of subjects.

In addition, a model was trained for each eye in current study instead of trying to train one model that can be applicable for all 17 eyes because of small dataset. Then, the repeatability of our 3D vitreous model has not been investigated well. Recent studies reported advanced U-Nets that enhance their performance configuring themselves and recovering lost image information^36,37^. Thus, our future goal is to create more generalized 3D models that can work for all eyes collecting more data.

In conclusion, we have presented a ML-based 3D modeling technique to examine the posterior vitreous cavities qualitatively and quantitatively. The vitreous cavity models showed good inter-examiner reliability and revealed that the shapes of the vitreous cavities are similar among schoolchildren. As liquefaction of vitreous gel progresses in young adulthood and degradation of the vitreous gel progresses due to aging, these 3D models will allow clinicians to determine the volume and surface area of vitreous fluid as new biometric parameters of the ocular conditions. Although the exact parameters have not been determined, we believe that this is a promising approach of detecting early changes caused by increasing age and eye diseases.

## Supplementary Information

Supplementary Video S1.

Supplementary Video S2.

Supplementary Information.

## Data Availability

The datasets analyzed during the current study are available from the corresponding author on reasonable request. Access to patient level data needs the approval of relevant data use agreements via Research Center for Industry Alliance, Tokyo Medical and Dental University.
