# Supplementary figures and images for: Machine learning-based 3D modeling and volumetry of human posterior vitreous cavity of optical coherence tomographic images
Source: Sci Rep. 2022 Aug 16;12:13836. doi: 10.1038/s41598-022-17615-z (PMC9381727; doi:10.1038/s41598-022-17615-z)

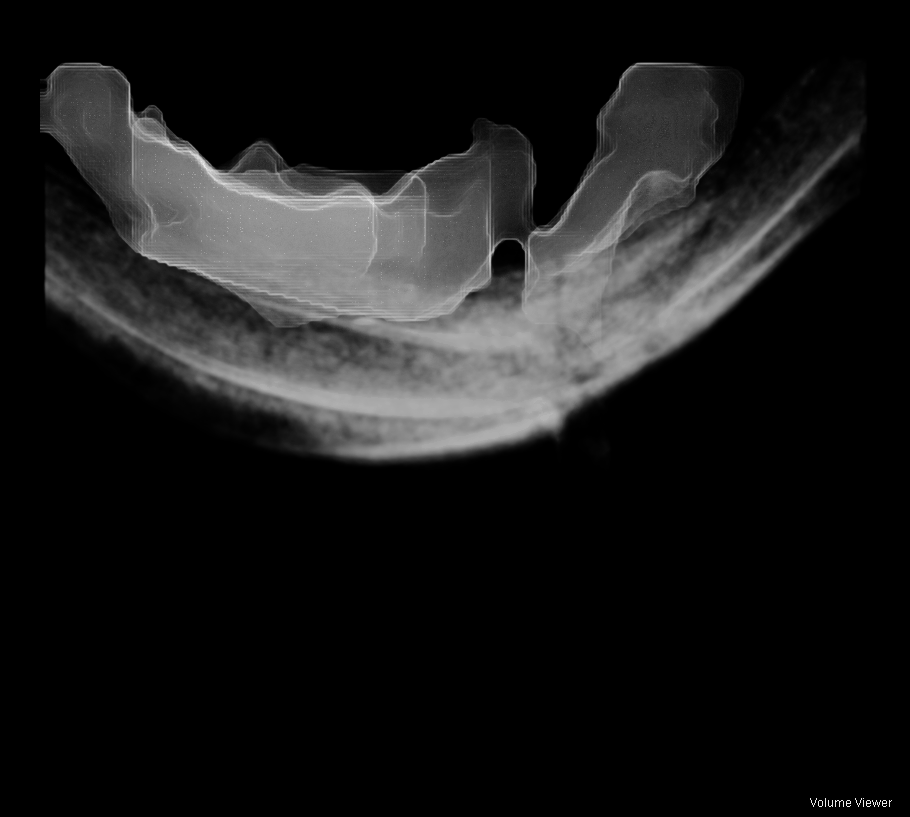

Supplement: Supplementary file 1 — Supplementary Video S1. [file 41598_2022_17615_MOESM1_ESM.gif]

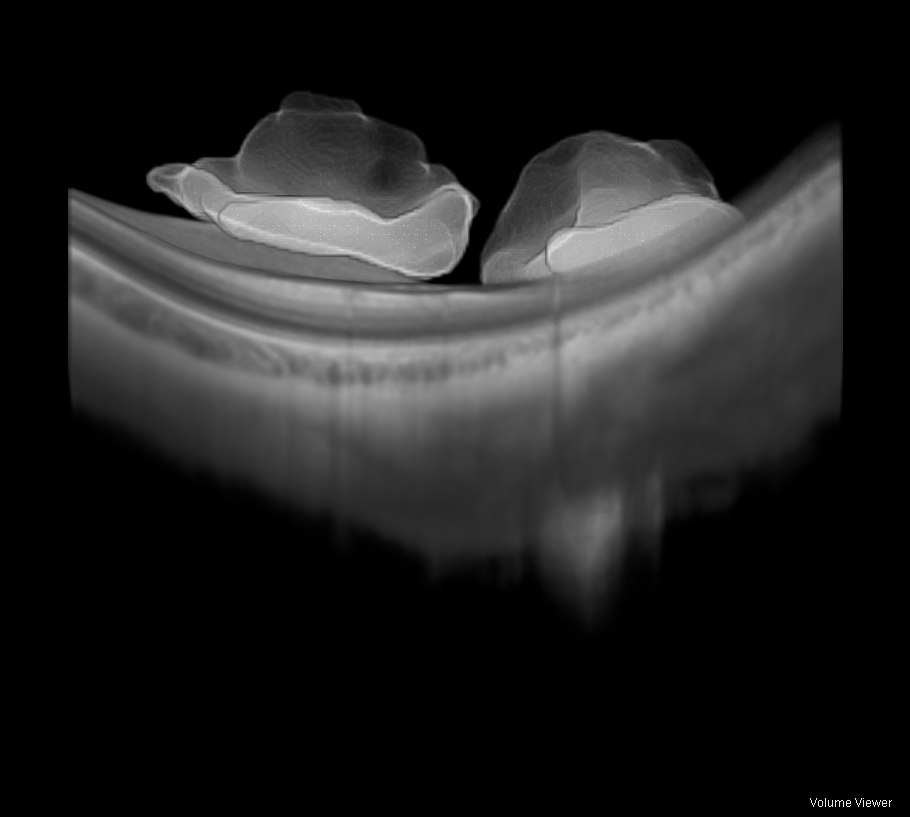

Supplement: Supplementary file 2 — Supplementary Video S2. [file 41598_2022_17615_MOESM2_ESM.gif]
