# Supplementary material for: Machine learning-based 3D modeling and volumetry of human posterior vitreous cavity of optical coherence tomographic images
Source: Sci Rep. 2022 Aug 16;12:13836. doi: 10.1038/s41598-022-17615-z (PMC9381727; doi:10.1038/s41598-022-17615-z)
Supplement: Supplementary file 3 — Supplementary Information. [file 41598_2022_17615_MOESM3_ESM.docx]

Supplementary Table 1. **Demographics of school children**

|  | Mean ± SD | Range |
| --- | --- | --- |
| Age (yrs) | 9.25 ± 1.39 | 7 to 12 |
| Sex (male/female) | 14 / 34 |  |
| Refractive error (diopters) | -3.54 ± 1.98 | +0.25 to -7.88 |
| Axial length (mm) | 24.59 ± 1.14 | 22.40 to 27.14 |

Plus-minus values are mean ± SD (standard deviation).

|  | Connection | | *P* value |
| --- | --- | --- | --- |
|  | Present | Absent |  |
| Number of eyes | 14 | 3 |  |
| Age (yrs) | 8.6 ± 1.2 | 8.0 ± 0.8 | 0.46* |
| Gender (male/female) | 4 / 10 | 1 / 2 | >0.99^†^ |
| Refractive error (diopter) | -2.06 ± 1.04 | -3.13 ± 0.81 | 0.14* |
| Axial length (mm) | 24.07 ± 0.78 | 25.04 ± 0.88 | 0.09* |
| Volume (mm^3^) |  |  |  |
| Whole cavities | 20.97 ± 7.87 | 13.33 ± 2.78 | 0.14* |
| Premacular vitreous pocket | 17.48 ± 7.77 | 9.52 ± 3.48 | 0.11* |
| Cloquet’s canal | 3.17 ± 1.34 | 2.28 ± 0.52 | 0.85* |
| Surface area (mm^2^) |  |  |  |
| Whole cavities | 108.64 ± 17.56 | 82.56 ± 3.61 | 0.03* |
| Premacular vitreous pocket | 87.50 ± 18.00 | 56.49 ± 9.21 | 0.02* |
| Cloquet’s canal | 22.73 ± 6.91 | 17.89 ± 4.19 | 0.99* |

Supplementary Table 2. **Comparison of Clinical and biometric data between eyes with and without connection between premacular vitreous pocket and Cloquet’s canal.**

Plus-minus values are mean ± SD (standard deviation). *Unpaired t test. †Fisher’s exact probability test.

**Supplemental Movie 1.** Three-dimensional movie of the vitreous model of the right eye of a healthy 8-year-old girl in Figure 2.

**Supplemental Movie 2.** Three-dimensional movie of the vitreous models of the right eye of a healthy 8-year-old boy in Figure 3.
